# The efficacy of the TEACH e-Learning course at improving early childhood educators’ physical activity and sedentary behaviour self-efficacy, knowledge, intentions, and perceived behavioural control: a randomized controlled trial

**DOI:** 10.1186/s12966-024-01628-0

**Published:** 2024-07-22

**Authors:** Matthew Bourke, Brianne A. Bruijns, Leigh M. Vanderloo, Jennifer Irwin, Rachel Heydon, Valerie Carson, Patti-Jean Naylor, Andrew M. Johnson, Kristi B. Adamo, Shauna M. Burke, Brian W. Timmons, Patricia Tucker

**Affiliations:** 1 School of Occupational Therapy, Faculty of Health Sciences, University of Western Ontario, 1201 Western Road, Elborn College, Room 2547, London, ON N6G 1H1 Canada; 2 ParticipACTION, Toronto, ON Canada; 3 School of Health Studies, Faculty of Health Sciences, University of Western Ontario, London, ON Canada; 4 Faculty of Education, University of Western Ontario, London, ON Canada; 5 Faculty of Kinesiology, Sport, and Recreation, University of Alberta, Edmonton, AB Canada; 6 School of Exercise Science, Physical and Health Education, University of Victoria, Victoria, BC Canada; 7 School of Human Kinetics, Faculty of Health Sciences, University of Ottawa, Ottawa, ON Canada; 8 Child Health and Exercise Medicine Program, McMaster University, Hamilton, ON Canada; 9 Children’s Health Research Institute, London, ON Canada

**Keywords:** Physical activity, e-Learning, Capacity building, Teacher training, Early childhood education, Randomized controlled trial

## Abstract

**Background:**

Early childhood educators play a critical role in promoting physical activity and reducing sedentary time in childcare centres. However, early childhood educators receive limited specialised pre- and in-service learning opportunities relating to these behaviours and may lack the capacity to effectively engage children in healthy movement behaviours. This study aimed to examine the efficacy of an e-Learning course on increasing early childhood educators’ physical activity and sedentary behaviour-related capacities.

**Methods:**

A two-group parallel randomized controlled trial was conducted with early childhood educators in Canada (M_age_ = 41.78, 97% female). Participants randomized to the intervention group were asked to complete a physical activity and sedentary behaviour e-Learning course within a 4-week period. Participants randomized to the waitlist control condition were assigned to a waitlist to receive the intervention after the testing period. Participants reported on their self-efficacy, knowledge, intentions, and perceived behavioural control relating to physical activity and sedentary behaviours at baseline, post-intervention, and 3 months follow-up. Linear mixed effects models were estimated to determine difference in changes in outcomes from baseline to post-intervention, and follow-up.

**Results:**

A total of 209 early childhood educators participated in the study (intervention *n* = 98; control *n* = 111). The TEACH e-Learning course was found to be efficacious at improving all of the examined outcomes, with standardized effect sizes ranging from d = 0.58 to d = 0.65 for self-efficacy outcomes, d = 0.66 to d = 1.20 for knowledge outcomes, d = 0.50 to d = 0.65 for intention outcomes, and d = 0.33 to d = 0.69 for perceived behavioural control outcomes post-intervention. The intervention effects were sustained at follow-up for all outcomes apart from perceived behavioural control to limit screen time. Additionally, the magnitude of the effect for knowledge outcomes decreased at follow-up, with standardized effect sizes ranging from d = 0.49 to d = 0.67.

**Conclusions:**

The e-Learning course was highly successful at improving early childhood educators’ capacity pertaining to physical activity and sedentary behaviours. Providing training content through e-Learning may be an efficacious approach to providing continual professional learning opportunities relating to physical activity and sedentary time to early childhood educators on a large scale.

**Supplementary Information:**

The online version contains supplementary material available at 10.1186/s12966-024-01628-0.

## Background

Regular engagement in physical activity and reducing the amount of time spent in sedentary screen-related activities are associated with a range of physical, mental, cognitive, and social health benefits in early childhood [1, 2]. Among the benefits associated with engaging in physical activity and limiting sedentary time include better body composition and bone health, fewer behavioural and emotional problems, and more positive social cognitive development [3]. Additionally, levels of physical activity and sedentary time in early childhood track moderately into later life, indicating that lifelong physical activity patterns may begin to develop from an early age [4]. Consequently, the World Health Organization [5] recommends that young children aged 1–4 years engage in at least 180 min of a range of physical activities at varying intensities each day, including at least 60 min of energetic play (moderate-to-vigorous intensity activity) for preschool-aged children (3–4 years). Additionally, it is recommended that children under 2 years of age do not participate in any sedentary screen time, and children aged 2–4 years engage in no more than 60 min of sedentary screen time each day [5].

Ensuring young children engage in sufficient levels of physical activity, and limit sedentary screen time is a public health priority [6]. Therefore, early childhood is a critical period to intervene to encourage young children to engage in healthy physical activity and sedentary behaviour patterns. Many children spend a large amount of time each day attending early childhood education settings making them a suitable setting to intervene. For example, in Canada 62% of children aged 1–3 years and 72% of children aged 4–5 years attended some form of childcare, the majority of whom were in centre-based childcare [7]. Inauspiciously, children have been found to spend more than 220 min of their time sedentary while at childcare and just 32 min engaged in moderate-to-vigorous physical activity [8], highlighting the need to better support the facilitation of physical activity opportunities in these environments.

Early childhood educators play a crucial role in promoting physical activity among young children through the practices they employ in childcare settings [9]. For example, early childhood educators can promote young children in their care to engage in more physical activity by role modelling active behaviours [10], providing opportunities for active play [11], teaching fundamental movement skills (e.g., jumping, throwing) [12], and scheduling time outdoors [13]. However, many early childhood educators receive limited or no specialised training relating to physical activity, outdoor play, or sedentary behaviours [14, 15]. Relatedly, early childhood educators regularly describe limited capacity, such as not having the necessary knowledge and practical skills, as major barriers to engaging young children in their care in physical activity [16]. Additionally, early childhood educators’ lack of physical activity-related self-confidence may decrease their motivation to increase children’s engagement in physical activity [16]. Indeed, multiple theoretical perspectives including the Social Cognitive Theory [17], and the Theory of Planned Behaviour [18] position perceptual factors at the forefront of predicting human behaviour, including self-efficacy, perceived behavioural control and intentions. Research has demonstrated that higher levels of self-efficacy among early childhood educators is related to greater physical activity policy adherence in early childhood educators [19], and that greater intentions from early childhood educators to engage children in physical activity is positively related to children’s physical activity levels while in childcare [20].

There is an obvious need to provide opportunities for early childhood educators to participate in professional learning related to physical activity and sedentary time to improve their capacity to support children in their care to be more physically active. Although several evidence-based strategies exist to increase physical activity in childcare settings [21], a lack of capacity may inhibit ECEs from selecting, adapting and implementing these strategies to improve the health of the children in their care [22]. Therefore, intervening to improve ECEs capacity may have wide ranging impacts on increasing children’s engagement in physical activity and has the potential for wide scale public health benefits [6]. Several studies have demonstrated the efficacy of in-person professional learning opportunities on physical activity related outcomes in early childhood educators [23–25]. A major advantage of face-to-face learning is the ability to have rich in-person interactions with peers and instructors in instructional environments which may support learners’ development through shared understanding [26, 27]. However, face-to-face learning has several disadvantages relating to accessibility and scalability such as resource constraints which limits the number of learners who can engage with the course at any given time (e.g., human resource and physical space constraints), a high level of inflexibility, and a high cost to implement [26]. For example, the cost of implementing a physical activity intervention for early childhood educators in Canada was estimated to be $350,000 annually, equal to about $285 per child in childcare centres participating in the intervention, and was mainly due to ongoing human resource costs [28]. On the other hand, e-Learning has several advantages including increased flexibility for learners to engage with content at their own pace and on their own time, improved accessibility to learning materials, and reduced ongoing costs [26, 29]. Therefore, implementing physical activity and sedentary behaviour-related professional learning through e-Learning courses could potentially provide increased accessibility to learning opportunities to a larger number of early childhood educators and with fewer ongoing costs.

Evidence from randomized controlled trials examining the efficacy of physical activity-related online professional learning courses for early childhood educators is limited and inconsistent. For example, in a small pilot randomized trial in the United States, researchers demonstrated that an online training course which included content relating to the importance of physical activity in early childhood, the role that ECEs play in promoting physical activity, and how to integrate structured physical activity into childcare improved early childhood educators’ physical activity-related knowledge, but was not efficacious at improving educators’ perceived capabilities or intentions to promote physical activity [30]. In a separate randomized controlled trial conducted in Australia, researchers demonstrated that an online training course consistent with the Social Cognitive Theory and including six modules relating to physical literacy (e.g., gross motor skills, active play, promoting motivation) increased early childhood educators’ physical literacy knowledge, competence, and confidence, but was not efficacious at changing their attitudes towards promoting physical activity or physical activity facilitation practices [31]. On the other hand, researchers from Canada showed that although an online training course related to both healthy eating and physical activity was not efficacious at improving early childhood educators’ fundamental movement skills and physical activity-related knowledge, it was efficacious at increasing their physical activity practices [32]. Still, there is more to be learned. Previous studies have some limitations that limit the generalizability of their results. These include employing ad hoc measures of educator outcomes without demonstrated validity; analyzing small sample sizes that were underpowered to detect an effect; and failing to include longer term follow-up assessments after the completion of the intervention.

Given the limited and inconsistent findings regarding the efficacy of physical activity-related online professional learning courses for early childhood educators, there is a need for more research in this area which addresses shortcomings of existing research. Results from the pilot study of the Training Early Childhood Educators in Physical Activity (TEACH) study demonstrated initial efficacy of the intervention among in-service early childhood educators in a single group study design [33] and showed that the intervention was highly acceptable and perceived to have an appropriate level of complexity to support learning [34]. The purpose of the current study was to build on the results of the TEACH pilot study by conducting a randomized control trial to examine the efficacy of an e-Learning course at increasing early childhood educators’ self-efficacy, intentions, and perceived behavioural control to increase physical activity and reduce sedentary time in childcare and improve early childhood educators’ physical activity and sedentary behaviour related knowledge compared to a control condition.

## Methods

### Study design and setting

This study is an extension of the original TEACH study, which was delivered to pre-service ECEs attending Canadian colleges. The study protocol for the original TEACH study has been published previously [35]. The outcomes and intervention in the current study are consistent with the pre-service TEACH study; however, the inclusion/exclusion criteria and participants recruitment differ in the current study (descried below), and the process evaluation described in the protocol was not conducted with in-service ECEs. The current study was a two-arm, parallel-group randomized controlled trial conducted between September 2022 and August 2023. Data collection was completed on three occasions, at baseline, post-intervention, and 3 months follow-up. On each occasion, participants were provided with a link to a Qualtrics questionnaire. Participants were asked to create a unique identification number to track their responses over multiple questionnaires. The methods and results from the TEACH randomized controlled trial were reported in accordance with the Consolidated Standards of Reporting Trials (CONSORT) [36]. Ethics approval for this study was received from the University of Western Ontario Non-Medical Research Ethics Board (project ID: 115,866).

### Participants and recruitment

Individuals were eligible to participate in this study if they were at least 18 years of age and a registered early childhood educator living and working in Canada at the time of recruitment. Participants also required access to the internet to complete the study assessments and engage in the intervention. Participants were recruited through online communications. For example, all members of the Canadian Child Care Federation were emailed about the study and invited to participate. Additionally, emails were sent directly to childcare centres to share the study details with their early childhood educators and the study was promoted on social media platforms (e.g., Facebook, Twitter, and Instagram). Participants who engaged in the study were eligible to enter the draw for a 1-in-10 chance to win an iPad.

### Sample size calculations

The required sample size was calculated in G*Power (v3.1.9.7). Based on results of the TEACH pilot study [33] and multiple other studies [23, 24], a moderate effect size was estimated for post-intervention and follow-up (d = 0.4). To achieve 80% power with an alpha of 0.05, a sample size of 200 participants was targeted.

### Randomization, allocation, and concealment

After completing the eligibility criteria questionnaire on Qualtrics (Provo, UT, USA), participants were randomized to either the intervention or waitlist control condition. Randomization on Qualtrics is automated with no input from researchers. After randomization, all participants were redirected to identical baseline questionnaires completed on Qualtrics and a page to provide their contact information. An email was sent to participants randomized to the intervention condition with a link to access the e-Learning course. Participants in the control condition were placed on a waitlist to access the e-Learning course at the conclusion of the study.

### Intervention

The TEACH intervention was a 4-module, 5-hour e-Learning course developed through a modified Delphi process which involved two expert panels of physical activity and sedentary behaviour researchers, and early childhood education experts in Canada [37]. The four modules covered: (1) an introduction to physical activity and sedentary behaviours in early childhood, including information on definitions, guidelines and recommendations, health benefits and consequences, and prevalence of behaviours; (2) factors influencing physical activity and sedentary time in the childcare environment, the importance of outdoor play, and how to encourage risky play; (3) strategies to promote physical activity and reduce sedentary time in childcare, such as goal setting, programming structured physical activities, role modelling behaviours, active learning, breaks and transitions, adapting the childcare environment to promote physical activity, creating policies, and partnering with families; and, (4) additional professional learning opportunities and a resource library. Consistent with Social Cognitive and Self-Efficacy theories [17, 38], the e-Learning content was designed to promote knowledge acquisition and improve participants’ perceptions of their capabilities to promote physical activity and reduce sedentary time. Therefore, the course incorporated several practical scenarios for early childhood educators to observe and scenario-based knowledge checks were implemented throughout the course. The e-Learning course utilized a combination of text, voiceover, videos, and animations to support participants’ learning. Participants were given up to 4 weeks to complete the e-Learning course. Participant progress through the e-Learning course was tracked to monitor intervention adherence, and reminder emails were sent after 2 weeks for participants who were yet to complete the course in an attempt to improve adherence. Additionally, knowledge checks were implemented to ensure that participants understood module content before moving onto the next module to ensure participant competence and understanding when completing the intervention.

### Primary outcome assessments

#### Self-efficacy

Participant’s physical activity and sedentary behaviour-related self-efficacy was assessed using the validated Early Childhood Educator Confidence in Outdoor Movement, Physical Activity, and Sedentary and Screen Behaviours (ECE-COMPASS) questionnaire [39]. The ECE-COMPASS questionnaire consists of 21 items to assess task self-efficacy (e.g., “how confident are you in your ability to create an environment that supports children’s active play?”) and 10 items to assess barrier self-efficacy (e.g., “how confident are you in your ability to encourage physical activity when [your] colleagues/superiors do not value it?”). Each item was scored on an 11-point Likert scale ranging from not confident at all (0) to completely confident (10). Participants’ task and barrier self-efficacy were calculated as their average response to each of the questions on each scale. Each of the scales had excellent internal consistently at baseline (McDonald’s ω [40] = 0.94–0.96), post-intervention (ω = 0.96–0.97), and follow-up (ω = 0.96–0.97).

#### Knowledge

A study-specific tool was developed to assess participants’ physical activity and sedentary behaviour-related knowledge specifically for this study. The tool included 8 items which assessed participants’ knowledge of *The Canadian 24-Hour Movement Guidelines for the Early Years* [41] and childcare-specific physical activity and screen-viewing recommendations (e.g., “how many minutes of moderate-to-vigorous physical activity (i.e., higher intensity physical activity) are preschoolers (3–4 years) recommended to engage in each day?”), 7 items which assessed knowledge of definitions (e.g., “what is an example of a muscle and bone-strengthening activity?”), and 7 items which assessed knowledge of favourable educator behaviours (e.g., “which of the following behaviours of early childhood educators does not promote physical activity?”). Participants were presented with four multiple choice options for each question as well as a fifth option to select if they did not know the answer. Composite scores were calculated as the number of questions participants answered correctly for each scale and overall.

### Secondary outcome assessments

#### Intentions

Intentions were assessed using the validated Early Childhood Educator Movement Behavioural Intention and Perceived Control (ECE-MBIPC) questionnaire [42]. The ECE-MBIPC questionnaire contains 7 subscales with 4 items each to assess intentions relating to engaging children in at least 120 min of physical activity each day (i.e., two-thirds of the recommended levels of physical activity across an entire day), promoting children’s development of physical literacy, being a good role model for children’s physical activity, minimizing long periods of sedentary time, avoiding children’s use of screen-based technology, promoting outdoor play during all seasons and weather conditions, and leading opportunities for outdoor risky play. Each intention subscale consisted of 4 items measured on a 7-point Likert scale and an overall score from 4 to 28 was calculated for each subscale. The subscales displayed acceptable-to-excellent internal reliability at baseline (ω = 0.70–0.86), post-intervention (ω = 0.83–0.94), and follow-up (ω = 0.87–0.95).

#### Perceived behavioural control

Perceived behavioural control was also assessed using the ECE-MBIPC questionnaire [42]. The perceived behavioural control questions from the ECE-MIBC contain the same content as the intention questions, however the stem of the questions are changed in order to assess perceived behavioural control. Each item was assessed on a 7-point Likert scale and an overall score from 4 to 28 was calculated for each subscale. The subscales displayed good-to-excellent internal reliability at baseline (ω = 0.88–0.93), post-intervention (ω = 0.84–0.93), and follow-up (ω = 0.88–0.93).

### Data analysis

Prior to data analysis, a missing value analysis was conducted to determine patterns of missing data. The missing value analysis indicated that participants with complete data reported significantly greater knowledge of definitions at baseline, t(206) = 2.79, *p* = .006. There were no significant differences between participants with complete and incomplete data on any other study or demographic variable, indicating that data were most likely missing completely at random. Missing data were accounted for in all analyses using restricted maximum likelihood estimations.

Data were analysed using the lme4 [43], lmerTest [44], and emmeans [45] packages in R (version 4.1.3). Linear mixed effects models including fixed effects of time (baseline, post-intervention, and follow-up), group (intervention and control), and a group-by-time interaction were estimated for all outcome variables. A significant group-by-time interaction (*p* < .05) indicates a significant intervention effect on the examined outcome. The models also included random intercepts to account for the clustering of repeated assessments within individual participants. Models were estimated using all available information using restricted maximum likelihood estimations. Residual plots from estimated models were inspected to determine if the assumption of homoscedasticity was violated. There was some evidence of heteroscedasticity; therefore, heteroscedastic robust standard errors were estimated for model parameters using the ClubSandwich package [46]. Standardized effect sizes were calculated for all effects at post-intervention and follow-up by dividing the mean difference by the pooled standard deviation at baseline (i.e., Cohen’s d). Standardized effect sizes were calculated to convey the practical significance of the results [47].

## Results

**Fig. 1:**
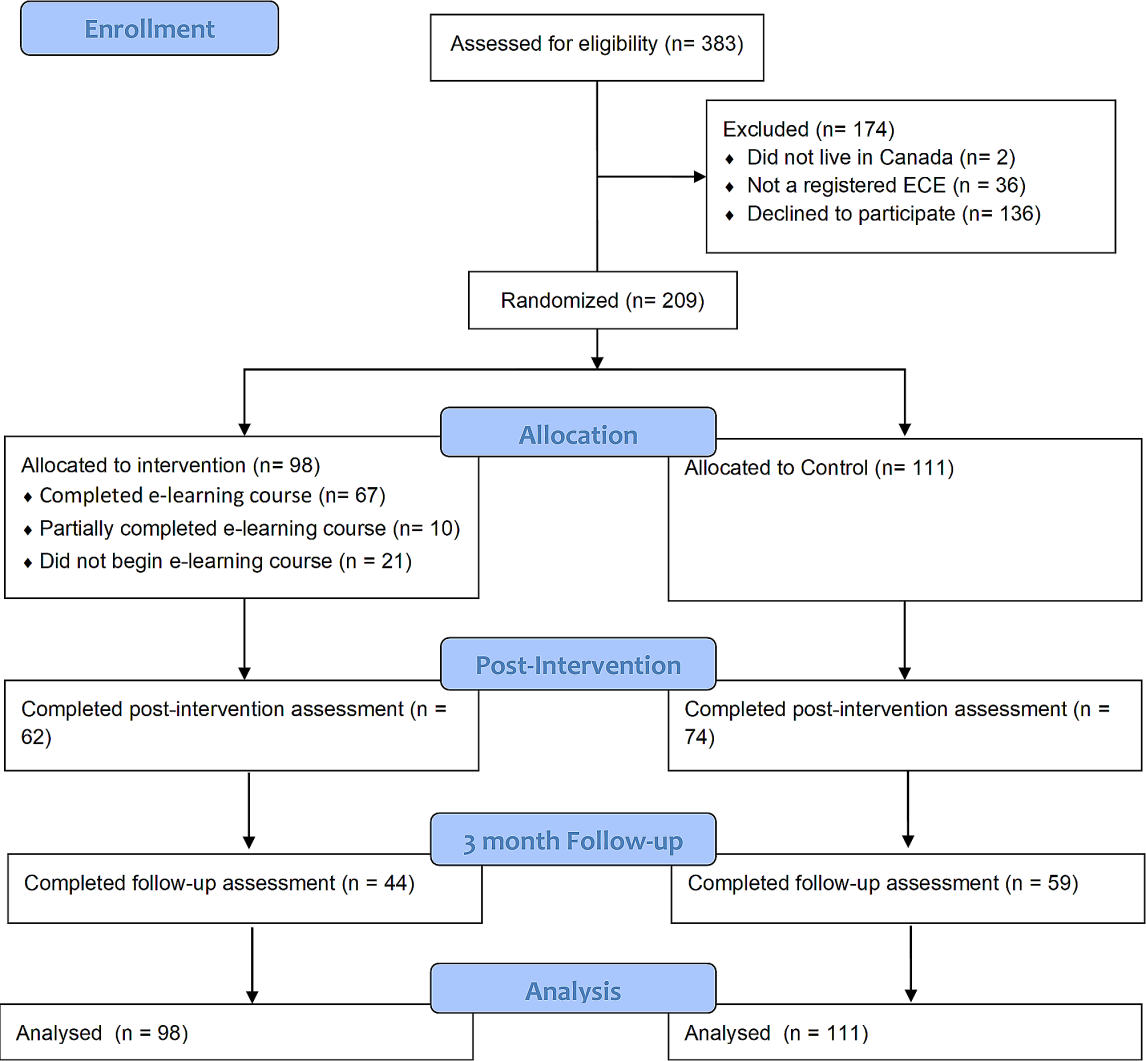
CONSORT participant flow chart

In total, 383 potential participants expressed interest in participating in the study and were assessed for eligibility. Of these, 174 participants were excluded. Thirty-eight did not meet the inclusion criteria and 136 did not complete the baseline assessment. Therefore, 209 participants who completed the baseline questionnaire were randomized to the intervention or waitlist control group (Fig. 1). Across the sample, participants were almost exclusively female (97.1%), had an average age of 41.78 years (*SD* = 11.28), were mainly white (66.3%), had received either a diploma (55.3%) or certificate (30.3%) in early childhood education, worked in centre-based childcare (66.3%), in preschooler (59.6%) and/or toddler (45.2%) classrooms, and had an average of 14.17 years (*SD* = 10.52) experience as an early childhood educator. The vast majority (71.6%) of participants did not engage in 150 min of physical activity each week; however, the majority (68.8%) of participants did limit their recreational screen time to less than 3 h per day. The descriptive statistics for participants in the intervention and waitlist control conditions are displayed in Table 1. Scores on each of the outcomes at baseline (see Appendix A) demonstrated that participants had relatively high levels of self-efficacy, perceived behaviour control, and intentions to promote physical activity and reduce screen time at baseline; however, their physical activity and sedentary behaviour related knowledge was limited, selecting the correct answer on only just over half of the knowledge questions at baseline.

**Table 1:** Participant characteristics

	Intervention	Control
Age M(SD)	43.02 (11.17)	40.70 (11.32)
Gender		
Female Male Non-binary Prefer not to answer	96 (98.0%) 2 (2.0%) 0 (0.0%) 0 (0.0%)	106 (96.4%) 1 (0.9%) 1 (0.9%) 2 (1.8%)
Ethnicity		
White Black East Asian Southeast Asian South Asian West Asian Arab Indigenous Other Prefer not to answer	62 (63.3%) 4 (4.1%) 7 (7.1%) 3 (3.1%) 5 (5.1%) 1 (1.0%) 2 (2.0%) 4 (4.1%) 2 (2.0%) 4 (4.1%)	76 (69.1%) 3 (2.7%) 2 (1.8%) 2 (1.8%) 7 (6.4%) 2 (1.8%) 1 (0.9%) 8 (7.3%) 3 (2.7%) 1 (0.9%)
Province		
British Columbia Alberta Saskatchewan Manitoba Ontario New Brunswick Nova Scotia Newfoundland and Labrador Prince Edward Island Northwest Territories Yukon	16 (16.3%) 20 (20.4%) 9 (9.2%) 16 (16.3%) 26 (26.5%) 2 (2.0%) 5 (5.1%) 4 (4.1%) 0 (0.0%) 0 (0.0%) 0 (0.0%)	20 (18.2%) 19 (17.3%) 15 (13.6%) 12 (10.9%) 24 (21.8%) 4 (3.6%) 9 (8.2%) 4 (3.6%) 1 (0.9%) 1 (0.9%) 1 (0.9%)
Type of Qualification		
Certificate Diploma Bachelor’s Degree Graduate Degree Other	29 (29.6%) 55 (56.1%) 5 (5.1%) 3 (3.1%) 6 (6.1%)	34 (30.9%) 60 (54.5%) 9 (8.2%) 3 (2.7%) 4 (3.6%)
Type of childcare facility		
Centre-based childcare Home-/family-based childcare Preschool Full-day kindergarten Other	64 (65.3%) 11 (11.2%) 8 (8.2%) 3 (3.1%) 6 (6.1%)	74 (67.3%) 8 (7.3%) 7 (6.4%) 5 (4.5%) 16 (14.5%)
Age group*		
Infants (0–1 years) Toddlers (1–2 years) Preschooler (2–4 years) Other	25 (25.5%) 46 (46.9%) 57 (58.2%) 5 (45.9%)	32 (29.1%) 48 (43.6%) 67 (60.9%) 47 (42.7%)
Years experiences M(SD)	15.58 (11.41)	12.92 (9.53)
Physical activity		
Less than 1 h/week 1–1.4 h/week 1.5–1.9 h/week 2–2.4 h/week 2.5 + hrs/week	22 (22.4%) 18 (18.4%) 10 (10.2%) 19 (19.4%) 29 (29.6%)	29 (26.4%) 23(20.9%) 12 (10.9%) 16 (14.5%) 30 (27.3%)
Recreational screen time		
Less than 1 h/day 1–1.9 h/day 2–2.9 h/day 3 + hrs/day	12 (12.2%) 31 (31.6%) 25 (25.5%) 30 (30.6%)	19 (17.3%) 26 (23.6%) 30 (27.3%) 35 (31.8%)
Previously completed an e-Learning course?		
Yes No	68 (69.4%) 30 (30.6%)	75 (68.2%) 35 (31.8%)

* Adds up to greater than 100% because participants could select multiple responses

### Primary outcomes

#### Changes in physical activity and sedentary behaviour-related self-efficacy

Changes in task and barrier self-efficacy for the intervention and waitlist control group are displayed in Table 2 and Appendix A. There was a significant intervention effect on task self-efficacy at post-intervention, d = 0.65, *p* < .001, which was sustained at 3-months follow-up, d = 0.62, *p* < .001. Similarly, there was a significant intervention effect on barrier self-efficacy, which increased significantly more in the intervention group from baseline to post intervention, d = 0.58, *p* < .001, which was sustained at 3 months follow-up, d = 0.64. *p* < .001.

**Table 2:** Results from mixed-effects models examining between-group differences in changes in self-efficacy, knowledge, intentions, and between the intervention and control group at post-intervention and follow-up

	Intervention		Control		Between group difference from baseline to post-intervention	d	*p*-value	Between group difference from baseline to 3 months follow-up	d	*p*-value
	Mean change from baseline to post-intervention	Mean change from baseline to 3 months follow-up	Mean change from baseline to post-intervention	Mean change from baseline to 3 months follow-up						
**Self-Efficacy**										
Task self-efficacy	1.18 (0.81, 1.56)	0.96 (0.51, 1.40)	0.14 (-0.21, 0.49)	-0.04 (-0.41, 0.33)	1.04 (0.62, 1.46)	0.65	< 0.001	1.00 (0.51, 1.48)	0.62	< 0.001
Barrier self-efficacy	1.21 (0.79, 1.62)	0.98 (0.49, 1.47)	0.18 (-0.21, 0.57)	-0.15 (-0.56, 0.26)	1.03 (0.59, 1.47)	0.58	< 0.001	1.13 (0.69, 1.67)	0.64	< 0.001
**Knowledge**										
Guideline knowledge	2.31 (1.76, 2.86)	1.22 (0.57, 1.86)	0.46 (-0.04, 0.96)	0.39 (-0.16, 0.94)	1.85 (1.28, 2.42)	1.09	< 0.001	0.82 (0.01, 1.63)	0.49	0.047
Definition knowledge	1.29 (0.90, 1.68)	0.56 (0.14, 1.05)	0.24 (-0.11, 0.60)	-0.09 (-0.48, 0.31)	1.05 (0.63, 1.48)	0.73	< 0.001	0.68 (0.11, 1.25)	0.48	0.019
Behavioural knowledge	1.06 (0.70, 1.42)	0.85 (0.43, 1.26)	0.19 (-0.14, 0.52)	0.10 (-0.25, 0.46)	0.87 (0.51, 1.22)	0.66	< 0.001	0.74 (0.29, 1.20)	0.56	0.002
Overall Knowledge	4.69 (3.97, 5.41)	2.58 (1.75, 3.41)	0.90 (0.24, 1.56)	0.46 (-0.26, 1.17)	3.79 (2.88, 4.70)	1.20	< 0.001	2.12 (0.84, 3.40)	0.67	0.001
**Intention**										
Program 120 min PA	2.70 (1.70, 3.71)	2.26 (1.08, 3.44)	0.09 (-0.84, 1.02)	-0.65 (-1.67, 0.36)	2.61 (1.52, 3.71)	0.54	< 0.001	2.91 (1.68, 4.15)	0.69	< 0.001
Promote physical literacy	2.12 (1.23, 3.01)	1.88 (0.83, 2.92)	0.04 (-0.78, 0.86)	-0.52 (-1.42, 0.37)	2.08 (1.09, 3.07)	0.60	< 0.001	2.40 (1.34, 3.47)	0.69	< 0.001
Role model PA	2.01 (1.19, 2.83)	1.92 (0.96, 2.88)	0.06 (-0.69, 0.81)	-0.38 (-1.20, 0.44)	1.96 (1.06, 2.86)	0.60	< 0.001	2.30 (1.23, 3.37)	0.71	< 0.001
Promote outdoor play	1.75 (0.85, 2.64)	1.78 (0.73, 2.83)	-0.58 (-1.41, 0.24)	-1.21 (-2.10, -0.31)	2.33 (1.58, 3.41)	0.60	< 0.001	2.98 (1.90, 4.07)	0.77	< 0.001
Lead risky play opportunities	2.36 (1.31, 3.41)	2.29 (1.06, 3.52)	-0.00 (-0.97, 0.96)	-0.88 (-1.93, 0.17)	2.36 (1.25, 3.48)	0.54	< 0.001	3.17 (1.84, 4.50)	0.73	< 0.001
Minimize sedentary time	2.32 (1.44, 3.20)	2.19 (1.16, 3.23)	0.01 (-0.81, 0.82)	-0.65 (-1.53, 0.24)	2.31 (1.27, 3.36)	0.65	< 0.001	2.84 (1.78, 3.90)	0.80	< 0.001
Avoid screen time	1.48 (0.40, 2.55)	1.37 (0.12, 2.62)	-0.39 (-1.38, 0.60)	-0.49 (-1.56, 0.58)	1.87 (0.58, 3.15)	0.50	0.005	1.86 (0.64, 3.08)	0.50	0.003
**Perceived Behaviour Control**										
Program 120 min PA	2.67 (1.49, 3.84)	1.91 (0.52, 3.29)	0.06 (-1.03, 1.16)	-0.42 (-1.75, 0.78)	2.60 (1.38, 3.81)	0.50	< 0.001	2.33 (0.78, 3.87)	0.45	0.004
Promote physical literacy	1.81 (0.90, 2.72)	1.62 (0.55, 2.69)	-0.16 (-1.00, 0.69)	-0.40 (-1.31, 0.51)	1.97 (1.02, 2.92)	0.53	< 0.001	2.02 (0.87, 3.17)	0.54	< 0.001
Role model PA	1.61 (0.75, 2.47)	1.21 (0.20, 2.23)	0.29 (-0.51, 1.09)	-0.59 (-1.46, 0.27)	1.32 (0.38, 2.25)	0.34	0.006	1.80 (0.73, 2.88)	0.47	0.001
Promote outdoor play	0.86 (-0.11, 1.83)	0.25 (-0.89, 1.39)	-0.64 (-1.54, 0.26)	-1.00 (-1.97, -0.02)	1.50 (0.38, 2.62)	0.36	0.009	1.25 (0.02, 2.47)	0.30	0.046
Lead risky play opportunities	1.85 (0.73, 2.98)	1.35 (0.03, 2.66)	-0.06 (-1.09, 0.98)	-0.28 (-1.40, 0.84)	1.91 (0.75, 3.07)	0.36	0.001	1.63 (0.19, 3.07)	0.31	0.027
Minimize sedentary time	2.38 (1.37, 3.38)	1.70 (0.52, 2.87)	-0.34 (-1.27, 0.60)	-0.67 (-1.68, 0.33)	2.72 (1.52, 3.92)	0.69	< 0.001	2.37 (1.10, 3.63)	0.60	< 0.001
Avoid screen time	0.93 (-0.10, 1.96)	0.37 (-0.83, 1.57)	-0.37 (-1.32, 0.58)	-0.16 (-1.19, 0.87)	1.30 (0.13, 2.47)	0.33	0.030	0.53 (-0.80, 1.86)	0.13	0.429

Note: Results presented as mean difference (95% confidence intervals)

#### Changes in physical activity and sedentary behaviour-related knowledge

Changes in knowledge for ECEs in the intervention and waitlist control groups are displayed in Table 2 and Appendix A. There was a significant and moderate-to-large intervention effect on guideline knowledge, d = 1.09, *p* < .001, knowledge of definitions, d = 0.73, *p* < .001, behavioural knowledge, d = 0.66, *p* < .001, and overall knowledge, d = 1.20, *p* < .001 at post intervention. The intervention had a significant effect on knowledge at 3-month follow-up; however, the magnitude of the effect was smaller than at post-intervention for knowledge of guidelines, d = 0.49, *p* = .047, knowledge of definitions, d = 0.48, *p* = .019, behavioural knowledge, d = 0.56, *p* = .002, and overall knowledge, d = 0.67, *p* = .001.

### Secondary outcomes

#### Changes in intentions to increase physical activity and decrease sedentary time

Changes in intentions for the intervention and waitlist control conditions are displayed in Table 2 and Appendix A. There was a significant intervention effect on intentions for all behaviours at post-intervention (*p* < .001 to *p* = .005), with standardized effect sizes ranging from d = 0.54 to d = 0.60. The intervention effect was sustained for all behavioural intentions at follow-up with increased standardized effect sizes ranging from d = 0.50 to d = 0.80.

#### Changes in perceived behavioural control to increase physical activity and decrease sedentary time

Changes in perceived behavioural control for the intervention and waitlist control conditions are displayed in Table 2 and Appendix A. There was a significant intervention effect on perceived behavioural control for all behaviours assessed at post-intervention with the standardized effect sizes ranging from d = 0.33 to d = 0.53. The significant intervention effect was sustained at follow-up for all forms of perceived behavioural control except for participants’ perceived behavioural control to avoid screen time for children in their care, d = 0.13, *p* = .429.

## Discussion

Findings from this randomized controlled trial demonstrated that the TEACH e-Learning course online training was highly efficacious at improving early childhood educators’ task and barrier self-efficacy and their knowledge relating to physical activity and sedentary time concepts. Additionally, the training was efficacious at increasing early childhood educators’ perceived behavioural control and intentions relating to physical activity, sedentary time, and outdoor and risky play.

Early childhood educators play a critical role in promoting young children’s engagement in healthy physical activity and sedentary behaviour patterns in childcare settings [9]. While early childhood educators recognise that they have a responsibility to promote physical activity and minimize screen-viewing among children in childcare [48], they cite a lack of specialised training as a major barrier limiting their ability to do so [16]. The perceived capabilities, knowledge, and intentions of early childhood educators to promote physical activity may all be related to the physical activity levels of children in their care [16, 19, 20]. Therefore, the results from the current study are highly encouraging and add to the growing body of literature demonstrating the potential efficacy of online training as an avenue to increase early childhood educator outcomes related to promoting physical activity and reducing sedentary time in childcare. Previous studies have demonstrated that online training may improve early childhood educators’ knowledge [30–32] and their competence and confidence [31]. The TEACH intervention is similar to previous interventions which were based on the Social Cognitive Theory [31] and included modules relating to the importance of physical activity, the role that ECEs play in promoting physical activity, and providing practical suggestions on ways that ECEs can increase physical activity in the childcare setting [30]. However, this study has some methodological strengths compared to previous studies including the use of validated assessments of outcomes, being sufficiently powered to examine a feasible effect size, and employing longer-term follow-up to assess the maintenance of the intervention effects, addressing important limitations in previous studies, and providing robust evidence. The results from the current study, with the evidence from previous studies, position evidence informed e-Learning courses, such as the TEACH course, as a feasible approach to improving early childhood educators’ capacity to promote physical activity and limit the amount of time children spend sedentary in childcare settings.

A promising finding from the study was that the intervention effects were sustained at 3-months follow-up, demonstrating that the TEACH e-Learning course may have both short-term and sustained effects. One possible explanation for this is that the e-Learning course contained several links to additional professional learning opportunities in the same topic area and included a resource library which early childhood educators could return to after completing the intervention to support their practices. However, similar to previous research showing that online training had a sustained effect on physical activity practices but not on knowledge [32], the results from the current study demonstrated that the effect of the intervention on knowledge outcomes was attenuated at follow-up. Therefore, it may be necessary to implement booster sessions or opportunities for ongoing and sustained learning opportunities to ensure that early childhood educators retain the knowledge of best practices and guidelines gained from the intervention.

Given the efficacy of the TEACH e-Learning course with regard to improving early childhood educator outcomes relating to physical activity and sedentary behaviours, and the relatively limited resources needed to implement the course at scale, the results from the present study suggest that there is potential to deliver robustly developed and evidence informed e-Learning courses as massive open online courses (MOOCs). MOOCs allow learners to access courses online, engage in self-regulated learning, and complete the courses at their own pace, in their own time, and in a place that is convenient for them, using multiple delivery formats (e.g., text, audio, video) [49]. Providing a physical activity and sedentary behaviour-related e-Learning course as a MOOC may be particularly useful to support the implementation of childcare physical activity and sedentary behaviour legislation or policies. Simply implementing new policy or legislation may not be effective at increasing physical activity in early childhood care settings [50]. However, coupling the implementation of new policies or legislation with capacity building can improve early childhood educators’ physical activity practices [51]. Physical activity and sedentary behaviour e-Learning courses may also be provided to support continuous professional learning among practicing early childhood educators and may be implemented into broader professional development programs or as part of accreditation standards. Using approaches consistent with those used in the TEACH course, such as incorporating several practical scenarios, implementing scenario-based knowledge checks, and sequentially delivering content in multiple formats (e.g., text, audio, image), with adaptations made to intervention content based local contexts, may be an efficacious approach to building early childhood educators’ capacity to promote physical activity and reduce sedentary time in childcare settings.

The current study has several strengths which may address some of the limitations of existing research. Specifically, the current study used multiple validated scales developed specifically to measure self-efficacy, perceived behavioural control, and intentions related to promoting physical activity and reducing sedentary time in childcare [39, 42]. Another major strength of the current study is the comprehensive methods used to develop the content of the TEACH e-Learning course. The content was developed through a rigorous Delphi process where over 60 early childhood education, physical activity and sedentary behaviour specialists from around the world were consulted to determine the content to be included in the e-Learning course [37]. Furthermore, results from the pilot study of the TEACH e-Learning course demonstrated that early childhood educators perceived the course as being highly acceptable and having an appropriate level of complexity to support learning, which may further explain its effectiveness [34]. The current study also included a 3-month follow-up assessment to demonstrate the sustained effect of the TEACH intervention.

Despite the strengths of the current study, there are also limitations, which need to be considered when interpreting the results. An important limitation to consider is the relatively high attrition rates. Specifically, only 63% and 45% of participants in the intervention group completed the post-intervention and follow-up assessments, respectively. Importantly, attrition rates were consistent across the intervention and waitlist control conditions. High levels of attrition are the norm rather than the exception in digital interventions [52]. For example, the median completion rates for MOOCs is estimated to be less than 15% [53]. Although modern missing data techniques can minimize the impact of missing data associated with attrition, biases may be introduced if the data is missing not at random [54]. Therefore, future e-Learning studies must consider how their interventions or data collection processes are designed to reduce attrition. Researchers may use concepts from persuasive system design such as task (e.g., reducing participant burden, tailoring intervention content based on participant responses), dialogue (e.g., providing positive feedback, sending constant reminders), system credibility (e.g., show that the intervention is based on expert knowledge), and social support (e.g., allowing participants to compare themselves to others) to improve adherence [55]. From a design perspective, researchers may employ a run-in period, where randomization takes place after enrolment in the study and all participants take part in a placebo intervention (e.g., an online module not related to the outcomes), and then only participants who complete the run in are randomized to either continue with a placebo or receive the intervention, effectively weeding out participants who are unlikely to adhere to the intervention [52, 56].

Several other limitations must also be considered. First, participants were not blinded and were aware of group allocation. Future research may benefit from the use of an attention control condition to ensure that participants are blinded to group assignment. Second, randomization occurred at the individual participant level. Therefore, it is possible that multiple ECEs working at the same childcare centre may have been randomized to different groups; therefore, contamination may have occurred for some participants in the waitlist control condition. Third, the knowledge questions were created specifically for this research study, and despite having face validity, the construct and concurrent validity of the knowledge questions is unknown, and the questions may not be generalizable. Additionally, the self-efficacy, perceived behavioural control, and intentions questions were self-report and, therefore, may be susceptible to response bias. Fourth, there was a relatively high attrition rate, especially at follow-up, with participants in the intervention group who completed the e-Learning course being more likely to complete follow-up assessments. Therefore, although all participants were included in the analysis through maximum likelihood estimations, the available data that the estimations were based upon were more likely to come from participants who completed the intervention. Consequently, the estimated intervention effect may be somewhat inflated. Fifth, the study focused on knowledge and perceptual outcomes, and did not consider child or teacher behavioural outcomes. Therefore, although knowledge and perceptual outcomes may be important predictors of ECE’s behaviours, it is not possible to determine if the intervention was effective at changing behavioural outcomes in practice from the results of the present study. Finally, the study only included English-speaking early childhood educators in Canada, and the participants in the study were highly experienced, with an average of over 12 years experience as an ECE. Additionally, there may have been a self-selection bias where ECEs interested in physical activity were more likely to participate in the study. Therefore, the results may not be generalizable to early childhood educators in other contexts or with less experience as an ECE.

## Conclusion

The results from this study demonstrated that robustly developed and evidence informed physical activity and sedentary behaviour focused e-Learning courses may be effective at improving early childhood educators’ physical activity and sedentary behaviour related self-efficacy, knowledge, intentions, and perceived behavioural control. These results provide support for using e-Learning as an approach to increase early childhood educators’ capacity to promote healthy movement behaviours in childcare settings. Given their potential for scalability, e-Learning courses could be delivered at a large scale to support the implementation of physical activity and sedentary time policies and legislation in childcare and be used to upskill early childhood educators through continuous professional learning.

### Electronic supplementary material

Below is the link to the electronic supplementary material.

Supplementary Material 1

## Data Availability

The datasets used for this current study are not publicly available, however will be made available upon reasonable request to the corresponding author pending ethics approval.

## Abbreviations

MOOC: Massive open online course
