# Supplementary material for: The efficacy of the TEACH e-Learning course at improving early childhood educators’ physical activity and sedentary behaviour self-efficacy, knowledge, intentions, and perceived behavioural control: a randomized controlled trial
Source: Int J Behav Nutr Phys Act. 2024 Jul 22;21:79. doi: 10.1186/s12966-024-01628-0 (PMC11265122; doi:10.1186/s12966-024-01628-0)
Supplement: Supplementary file 1 — Supplementary Material 1 [file 12966_2024_1628_MOESM1_ESM.docx]

Table A1 – Detailed summary of results

|  | Intervention | | | | | Control | | | | | Between group difference from baseline to post | p-value | Between group difference from baseline to follow-up | p-value |
| --- | --- | --- | --- | --- | --- | --- | --- | --- | --- | --- | --- | --- | --- | --- |
|  | Baseline | Post | Follow-up | Mean change from baseline to post | Mean change from baseline to follow-up | Baseline | Post | Follow-up | Mean change from baseline to post | Mean change from baseline to follow-up |  |  |  |  |
| **Self-Efficacy** |  |  |  |  |  |  |  |  |  |  |  |  |  |  |
| Task self-efficacy | 7.60  (7.28, 7.91) | 8.78  (8.42, 9.14) | 8.55  (8.14, 8.96) | 1.18  (0.81, 1.56) | 0.96  (0.51, 1.40) | 7.76  (7.47, 8.06) | 7.91  (7.57, 8.25) | 7.72  (7.37, 8.08) | 0.14  (-0.21, 0.49) | -0.04  (-0.41, 0.33) | 1.04  (0.62, 1.46) | <.001 | 1.00  (0.51, 1.48) | <.001 |
| Barrier self-efficacy | 7.46  (7.10, 7.81) | 9.67  (8.26, 9.07) | 8.44  (7.98, 8.90) | 1.21  (0.79, 1.62) | 0.98  (0.49, 1.47) | 7.46  (7.12, 7.79) | 7.63  (7.25, 8.02) | 7.31  (6.91, 7.71) | 0.18  (-0.21, 0.57) | -0.15  (-0.56, 0.26) | 1.03  (0.59, 1.47) | <.001 | 1.13  (0.69, 1.67) | <.001 |
| **Knowledge** |  |  |  |  |  |  |  |  |  |  |  |  |  |  |
| Guideline knowledge | 2.63  (2.29, 2.97) | 4.94  (4.52, 5.36) | 3.85  (3.34, 4.35) | 2.31  (1.76, 2.86) | 1.22  (0.57, 1.86) | 2.39  (2.07, 2.71) | 2.85  (2.47, 3.23) | 2.78  (2.35, 3.21) | 0.46  (-0.04, 0.96) | 0.39  (-0.16, 0.94) | 1.85  (1.28, 2.42) | <.001 | 0.82  (0.01, 1.63) | .047 |
| Definition knowledge | 4.68  (4.40, 4.96) | 5.97  (5.64, 6.31) | 5.28  (4.89, 5.66) | 1.29  (0.90, 1.68) | 0.56  (0.14, 1.05) | 4.79  (4.53, 5.06) | 5.03  (4.73, 5.34) | 4.71  (4.37, 5.04) | 0.24  (-0.11, 0.60) | -0.09  (-0.48, 0.31) | 1.05  (0.63, 1.48) | <.001 | 0.68  (0.11, 1.25) | .019 |
| Behavioural knowledge | 5.18  (4.94, 5.41) | 6.24  (5.94, 6.53) | 6.02  (5.68, 6.36) | 1.06  (0.70, 1.42) | 0.85  (0.43, 1.26) | 5.25  (5.02, 5.47) | 5.44  (5.17, 5.70) | 5.35  (5.06, 5.64) | 0.19  (-0.14, 0.52) | 0.10  (-0.25, 0.46) | 0.87  (0.51, 1.22) | <.001 | 0.74  (0.29, 1.20) | .002 |
| Overall Knowledge | 12.48  (11.86, 13.11) | 17.17  (16.42, 17.92) | 15.05  (14.21, 15.92) | 4.69  (3.97, 5.41) | 2.58  (1.75, 3.41) | 12.44  (11.85, 13.02) | 13.33  (12.64, 14.02) | 12.89  (12.15, 13.64) | 0.90  (0.24, 1.56) | 0.46  (-0.26, 1.17) | 3.79  (2.88, 4.70) | <.001 | 2.12  (0.84, 3.40) | .001 |
| **Intention** |  |  |  |  |  |  |  |  |  |  |  |  |  |  |
| Program 120 minutes PA | 22.86  (22.07, 23.65) | 25.57  (24.63, 26.50) | 25.12  (24.06, 26.18) | 2.70  (1.70, 3.71) | 2.26  (1.08, 3.44) | 24.10  (23.35, 24.84) | 24.19 (23.32, 25.05) | 23.44  (22.51, 24.37) | 0.09  (-0.84, 1.02) | -0.65  (-1.67, 0.36) | 2.61  (1.52, 3.71) | <.001 | 2.91  (1.68, 4.15) | <.001 |
| Promote physical literacy | 24.03  (23.39,  24.68) | 26.15  (25.39, 26.92) | 25.91  (25.02, 26.80) | 2.12 (1.23, 3.01) | 1.88  (0.83, 2.92) | 24.63  (24.03, 25.23) | 24.67  (23.96, 25.38) | 24.11  (23.34, 24.87) | 0.04  (-0.78, 0.86) | -0.52  (-1.42, 0.37) | 2.08  (1.09, 3.07) | <.001 | 2.40  (1.34, 3.47) | <.001 |
| Role modelling PA | 24.22 (23.61, 24.85) | 26.23  (25.51, 26.95) | 26.14  (25.31, 26.97) | 2.01 (1.19, 2.83) | 1.92  (0.96, 2.88) | 24.99 (24.43, 25.56) | 25.05  (24.39, 25.72) | 24.61  (23.89, 25.33) | 0.06  (-0.69, 0.81) | -0.38  (-1.20, 0.44) | 1.96  (1.06, 2.86) | <.001 | 2.30  (1.23, 3.37) | <.001 |
| Promote outdoor play | 24.26  (23.53, 24.99) | 26.00  (25.16, 26.85) | 26.03  (25.08, 26.99) | 1.75  (0.85, 2.64) | 1.78  (0.73, 2.83) | 25.38  (24.69, 26.06) | 24.79  (24.01, 25.58) | 24.17  (23.33, 25.01) | -0.58  (-1.41, 0.24) | -1.21  (-2.10, -0.31) | 2.33  (1.58, 3.41) | <.001 | 2.98  (1.90, 4.07) | <.001 |
| Lead risky play opportunities | 23.14  (22.28, 23.99) | 25.50  (24.51, 26.49) | 25.43  (24.31, 26.55) | 2.36  (1.31, 3.41) | 2.29  (1.06, 3.52) | 23.52  (22.72, 24.32) | 23.51  (22.60, 24.43) | 22.64  (21.66, 23.62) | -0.00  (-0.97, .96) | -0.88  (-1.93, 0.17) | 2.36  (1.25, 3.48) | <.001 | 3.17  (1.84, 4.50) | <.001 |
| Minimize sedentary time | 23.75  (23.11, 24.38) | 26.07  (25.30, 26.83) | 25.94  (25.06, 26.82) | 2.32  (1.44, 3.20) | 2.19  (1.16, 3.23) | 24.71  (24.11, 25.31) | 24.71  (24.01, 25.42) | 24.06  (23.30, 24.82) | 0.01  (-0.81, 0.82) | -0.65  (-1.53, 0.24) | 2.31  (1.27, 3.36) | <.001 | 2.84  (1.78, 3.90) | <.001 |
| Avoid screen time | 25.14  (24.45, 25.82) | 26.61  (25.77, 27.46) | 26.50  (25.51, 27.50) | 1.48  (0.40, 2.55) | 1.37  (0.12, 2.62) | 25.94 (25.31, 26.58) | 25.55  (24.78, 26.33) | 25.45  (24.60, 26.31) | -0.39  (-1.38, 0.60) | -0.49  (-1.56, 0.58) | 1.87  (0.58, 3.15) | .005 | 1.86  (0.64, 3.08) | .003 |
| **Perceived Behaviour Control** |  |  |  |  |  |  |  |  |  |  |  |  |  |  |
| Program 120 minutes PA | 22.18 (21.18, 23.18) | 24.84 (23.69, 25.99) | 24.08 (22.78, 25.38) | 2.67 (1.49, 3.84) | 1.91 (0.52, 3.29) | 23.07 (22.13, 24.01) | 23.14, 22.06, 24.22) | 22.66 (21.51, 23.80) | 0.06  (-1.03, 1.16) | -0.42  (-1.75, 0.78) | 2.60  (1.38, 3.81) | <.001 | 2.33  (0.78, 3.87) | .004 |
| Promote physical literacy | 23.88 (23.15, 24.61) | 25.67 (24.84, 26.53) | 25.50 (24.53, 26.46) | 1.81 (0.90, 2.72) | 1.62 (0.55, 2.69) | 24.14 (23.46, 24.82) | 23.98 (23.19, 24.77) | 23.74 (22.90, 24.58) | -0.16  (-1.00, 0.69) | -0.40  (-1.31, 0.51) | 1.97  (1.02, 2.92) | <.001 | 2.02  (0.87, 3.17) | <.001 |
| Role modelling PA | 24.26 (23.54, 24.97) | 25.87 (25.04, 26.69) | 25.47 (24.53, 26.41) | 1..61 (0.75, 2.47) | 1.21 (0.20, 2.23) | 24.74 (24.07, 25.41) | 25.03 (24.26, 25.80) | 24.15 (23.33, 24.97) | 0.29  (-0.51, 1.09) | -0.59  (-01.46, 0.27) | 1.32  (0.38, 2.25) | .006 | 1.80  (0.73, 2.88) | .001 |
| Promote outdoor play | 24.21 (23.35, 25.07) | 25.07  (24.10, 26.05) | 24.46  (23.37, 25.55) | 0.86  (-0.11, 1.83) | 0.25  (-0.89, 1.39) | 24.59 (23.79, 25.39) | 23.96 (23.05, 24.86) | 23.60 (22.63, 24.56) | -0.64  (-1.54, 0.26) | -1.00  (-1.97, -0.02) | 1.50  (0.38, 2.62) | .009 | 1.25  (0.02, 2.47) | .046 |
| Lead risky play opportunities | 23.03 (22.00, 24.05) | 24.88  (23.73, 26.03) | 24.37 (23.09, 25.65) | 1.85 (0.73, 2.98) | 1.35  (0.03, 2.66) | 22.69  (21.74, 23.63) | 22.63 (21.56, 23.70) | 22.40 (221.27, 23.54) | -0.06  (-1.09, 0.98) | -0.28 (-1.40, 0.84) | 1.91  (0.75, 3.07) | .001 | 1.63  (0.19, 3.07) | .027 |
| Minimize sedentary time | 23.80 (23.05, 24.56) | 26.18 (25.29, 27.07) | 25.50  (24.78, 26.52) | 2.38  (1.37, 3.38) | 1.70  (0.52, 2.87) | 24.43  (23.73, 25.13) | 24.09  (23.26, 24.91) | 23.76  (22.87, 24.64) | -0.34  (-1.27, 0.60) | -0.67  (-1.68, 0.33) | 2.72  (1.52, 3.92) | <.001 | 2.37  (1.10, 3.63) | <.001 |
| Avoid screen time | 25.46  (24.71, 26.21) | 26.38  (25.49, 27.27) | 25.83  (24.80, 26.85) | 0.93  (-0.10, 1.96) | 0.37  (-0.83, 1.57) | 25.69 (25.00, 26.39) | 25.32  (24.50, 26.15) | 25.53  (24.64, 26.42) | -0.37  (-1.32, 0.58) | -0.16  (-1.19, 0.87) | 1.30  (0.13, 2.47) | .030 | 0.53  (-0.80, 1.86) | .429 |
